# Understanding Patient Experiences to Inform Future Studies to Optimize Personalization of Treatment for Early Breast Cancer

**DOI:** 10.1245/s10434-024-15459-7

**Published:** 2024-05-21

**Authors:** Stuart A. McIntosh, Mhairi Mactier, Katherine Fairhurst, Jacqui Gath, Hilary Stobart, Shelley Potter

**Affiliations:** 1 Patrick G. Johnston Centre for Cancer Research, Queen’s University Belfast, Belfast, UK; 2 General Surgery Department, Golden Jubilee National Hospital, Glasgow, UK; 3 Centre for Surgical Research, Department of Population Health Sciences, Bristol Medical School, University of Bristol, Bristol, UK; 4 Independent Cancer Patients’ Voice, London, UK; 5 Bristol Surgical and Perioperative Care Complex Intervention Collaboration, Bristol Medical School, Bristol, UK; 6 Bristol Breast Care Centre, North Bristol NHS Trust, Bristol, UK

**Keywords:** Breast cancer, Personalized treatment, Treatment toxicity, Patient experience, Patient survey

## Abstract

**Background:**

Breast cancer treatment is multimodal, but not all patients benefit from each treatment, and many experience morbidities significantly impacting quality of life. There is increasing interest in tailoring breast cancer treatments to optimize oncological outcomes and reduce treatment burden, but it is vital that future trials focus on treatments that most impact patients. This study was designed to explore patient experiences of treatment to inform future research.

**Methods:**

An online survey was co-developed with patient advocates to explore respondents’ experiences of breast cancer treatment. Questions included simple demographics, treatments received, and views regarding omitting treatments if that is deemed safe. The survey was circulated via social media and patient advocacy groups. Responses were summarized by using simple statistics; free text was analyzed thematically.

**Results:**

Of the 235 participants completing the survey, 194 (82.6%) would choose to omit a specific treatment if safe to do so. The most commonly selected treatments were chemotherapy (*n* = 69, 35.6%) and endocrine therapy (*n* = 61, 31.4%) mainly due to side effects. Fewer respondents would choose to omit surgery (*n* = 40, 20.6%) or radiotherapy (*n* = 20, 10.3%). Several women commented that survival was their “absolute priority” and that high-quality evidence to support the safety of reducing treatment would be essential.

**Conclusions:**

Patients with breast cancer are individuals who may wish to optimize different components of their treatment. A portfolio of studies co-designed with patients is needed to establish an evidence base for greater treatment personalization with studies focused on reducing avoidable chemotherapy and endocrine therapy a priority.

Breast cancer is the most common cancer in the United Kingdom with approximately 55,500 new cases diagnosed each year.^1^ Outcomes for women with early breast cancer have improved significantly over the past few decades, with overall 10-year survival of more than 75% in the United Kingdom.^1^ For smaller, good prognosis tumours, breast cancer-specific survival now approaches 100% at 10 years.^2^

Treatment of breast cancer remains multimodal. Most patients have surgery, radiotherapy, and endocrine therapy. A significant proportion also are given adjuvant systemic therapies, such as cytotoxic chemotherapy or anti-HER2 treatment. This approach is largely standardised, but it is increasingly recognised that not all patients need, or will benefit from, some of the treatments received. This may be because of the low-risk nature of their disease or because the biology of their disease means that some treatments are ineffective. Most, however, will experience side effects and toxicities that will adversely impact their quality of life and well-being. Furthermore, the often long-term and ongoing provision of potentially harmful treatments with minimal benefit has significant cost implications for healthcare providers.

There is a need for a more personalised approach to breast cancer such that patients receive only effective components of treatment, maintaining excellent oncological outcomes while deescalating or omitting minimally beneficial components, reducing treatment burden and related morbidity, and improving quality of life. Indeed, several of the recently identified top ten UK research priorities for breast cancer surgery focus on optimising aspects of treatment to reduce morbidity, highlighting the importance of this area to both patients and clinicians.^3^ The provision of optimised or risk-adapted breast cancer treatment is already the subject of multiple studies employing various strategies, including gene expression profiling or other biomarkers or imaging, to select patients for safe omission of adjuvant treatments, including chemotherapy, radiation therapy, and endocrine therapy.^4–9^ While these studies are welcome and have been robustly designed in conjunction with patient partners, little work to date has focused on exploring how different treatments impact patients and which components of treatment patients themselves may choose to omit if it is safe. Such work is key as clinician/researcher and patient views on treatment impact may differ. Indeed, although a large number of recent and ongoing studies focus on the safe omission of radiotherapy, a Canadian survey of 130 patients reported that most women would prefer to receive radiotherapy rather than endocrine therapy given that endocrine therapy has a greater impact on quality of life.^7,9–11^

Understanding patient experiences is vital to ensure that future trials are designed to address questions that are the most important and meaningful to breast cancer patients. This study surveyed individuals with lived experience of early breast cancer to better understand their experiences of treatment to support the development of future patient-centred, risk-adapted studies designed to optimize breast cancer treatment outcomes.

## Methods

An online survey was co-developed with patient advocates using REDCap data management software to explore respondents’ experiences of treatments for early breast cancer.^12^ Questions included simple demographics, details of treatments received, and views about omitting therapies. Free-text boxes were provided for respondents to provide additional information about their choices. The survey was piloted and iteratively refined before the launch to ensure that it was clear and comprehensible to potential respondents.

Individuals with personal experience of treatment for early breast cancer were invited to complete the survey via social media, patient advocacy groups, and breast cancer patient networks worldwide between April 1, 2023 and August 31, 2023.

Responses were summarised by using simple descriptive statistics, and free text was analysed thematically.^13^ Full ethical approval for the project was obtained from Queen’s University Belfast Faculty of Medicine, Health and Life Sciences (reference MHLS 23_17) before participant recruitment.

## Results

A total of 235 women with lived experience of treatment for breast cancer completed the survey. Respondent demographics are summarized in Table 1. Most were white (*n* = 225, 95.7%) and between 40 and 60 years of age (*n* = 158, 67.2%). Almost 60% (*n* = 134, 57.0%) lived in the United Kingdom, but there was broad geographical spread of respondents from Europe and North America. The median year of diagnosis was 2018 (range 1989–2023). The majority of respondents (*n* = 211, 89.8%) had undergone surgery as part of their breast cancer treatment. Approximately two thirds received endocrine therapy (*n* = 158, 67.2%) and/or radiotherapy (*n* = 150, 63.8%), and 60% (*n* = 139, 139, 59.1%) had chemotherapy. Fewer respondents reported receiving anti-HER-2–directed therapy, CDK 4/5 inhibitors, or other treatments (Table 1).

**Table 1:** Demographics of survey respondents

	*N* = 235 (%)
Age at diagnosis	
≤ 40	35 (14.9)
40–50	79 (33.6)
50–60	79 (33.6)
60–70	33 (14.0)
> 70	5 (2.1)
Not stated	4 (1.7)
Year of diagnosis	
Before 2000	7 (3.0)
2000–2005	9 (3.8)
2006–2010	15 (6.4)
2011–2015	22 (9.4)
2016–2020	53 (22.6)
2021–2023	53 (22.6)
Not stated	79 (32.3)
Ethnicity	
White	225 (95.7)
Other/not stated	10 (4.3)
Country of residence	
United Kingdom	134 (57.0)
North America	29 (12.3)
Europe	12 (5.1)
Other	2 (0.9)
Not stated	58 (24.7)
Breast cancer treatments received	
Surgery	211 (89.8)
Radiotherapy	150 (63.8)
Chemotherapy	139 (59.1)
Anti-HER2 therapy	36 (15.3)
Endocrine therapy	158 (67.2)
CDK4/6 inhibitor	20 (8.5)
Other treatment^a^	37 (15.7)
Treatment would like to omit if safe to do so	*N* = 194*
Chemotherapy	69 (35.6)
Endocrine therapy	61 (31.4)
SurgeryRadiotherapy	40 (20.6)20 (10.3)
Other treatment^b^	4 (2.1)

^a^Including immunotherapy, bisphosphonates, demosumab and other targeted therapies

^b^Bisphosphonates *n* = 1, immunotherapy *n* = 1, anti-HER-2 therapy *n* = 1, CDK 4/6 inhibitor *n* = 1

*Participants with experience of early breast cancer treatment who expressed a preference regarding treatment omission

### Preferences for Safe Omission of Treatments

Of the 235 respondents, 194 (82.6%) women with experience of treatment for early breast cancer expressed a preference regarding omitting a component of their treatment if it had been safe to do so. The most commonly chosen treatment was chemotherapy (n = 69, 35.6%) followed by endocrine therapy (n = 61, 31.4%) and surgery (*n* = 40, 20.6%). Only 10% (*n* = 20) of women would have opted to omit radiotherapy (Table 1).

### Rationale for Omitting Treatments

Respondents’ rationale for wishing to omit specific treatments are summarized in Table 2.

**Table 2:** Reasons for omission of treatment

*Key themes*	*Example respondent quotes*
*Reasons for omitting chemotherapy*	
Short- and long-term side effects of treatment	*‘The chemotherapy made me feel terrible for about a week or more, starting 2–3 days after receiving each treatment (6 total). I had nausea and diarrhoea and food tasted bad and felt weird in my mouth. I had sores in my mouth and at the corners of my lips that wouldn’t heal. It was definitely the most terrible part of my cancer treatment.”*
	*‘‘Now I am left with peripheral neuropathy that effects my activities of daily living, e.g., fingers and feet numb. Also, my energy levels still drop quite dramatically, and I have to manage my commitments very carefully.”*
Impact of short-term side effects on day-to-day activities, lifestyle, family, and ability to work	*“Baldness and sepsis from chemo very impactful on my ability to work and care for my young child.”*
	*“The side effects were horrible, and the stress of the mental and physical toil of chemotherapy put me and my family under huge strain.”*
Psychological impact of treatment	*“I felt it was the chemo that made me sick.... I hated when my hair fell out and then I became a ‘cancer patient.’ I hated that everyone knew I was sick.”*
	*“I felt I lost my identity during chemo; psychologically it was so tough to go to treatment feeling sort of ok, knowing that after treatment I’d be back to feeling rubbish.”*
*Reasons for omitting endocrine therapy*	
Side effects, including joint pain, fatigue, hot flushes, weight gain, thinning of hair	*“It affects me every day. Always too hot, so doing sports is very uncomfortable now. Plus disruption of sleep and weight gain.”*
	*“It changed the way I looked and felt within a few months of starting it. I suddenly looked old and coarse. I felt stiff and slow.”*
Long-term impact of treatment on well-being, quality of life, and mental health	*“Endocrine therapy seems to be a 10-year sentence, which makes every day miserable; existing not living.”*
	*“Long-term quality-of-life side effects, destroying who I was, such a long time…I will be 60 when treatment finishes. Disturbed sleep, hot flashes, and fatigue are debilitating.”*
	*“The endocrine treatment ruined my sex life. Not only did it reduce my interest but my vagina shrunk so much that I cannot even get my own finger in, let alone a penis!!”*
Daily reminder of having had breast cancer	*“Taking a daily tablet means it’s a daily reminder you’ve had cancer.”*
Lack of long-term support for management of side effects of endocrine therapy	*“I feel that there needs to be an understanding of and sympathy towards the knock on effects of treatment. I had a chemical menopause at 33 and have found managing two young children and a full-time job to be really hard whilst dealing with anxiety, sweats, insomnia, joint pain, etc. There has been a general sense that this is the new normal, and I have to get on with it, but it is debilitating.”*
	*“Hormone treatment also has significant and ongoing side effects but with much less support than other treatment modalities. A feeling that treatment is finished despite ongoing hormone treatment and side effects, more difficult to access support, ask questions, have symptoms managed.”*
*Reasons for omitting surgery*	
Impact on body image	*“My breast is deformed now.”*
	*“The most disfiguring.”*
Long-term psychological impact	*“I feel like this has the greatest long-term effects, particularly psychologically.”*
	*“It resulted in a radical change of lifestyle and affected every aspect of my life.”*
Need for multiple operations	*“Having to go through three operations.”*
Recovery and complications of surgery	*“It was the most uncomfortable and disruptive treatment of them all. I had to leave my home for two nights, and after I’d got used to that they nearly sent me home with drains, which stressed me. Then, I swelled up and had to go back three times for drainage.”*
*Reasons for avoiding specific procedures: Mastectomy ± breast reconstruction*	
Psychological impact of losing a breast	*“Mentally more impact than physically. I have been left feeling abnormal and half a woman.”*
	*“Losing a breast even though I opted to have an immediate reconstruction…it’s very traumatic losing a breast.”*
	*“I would have done anything to avoid the mastectomy. Worst experience of my life and totally life-changing in a very negative way.”*
Impact on intimacy and relationship with partner	*“I feel ugly after my mastectomy; it is still painful to touch. It has affected my sex life with my husband.”*
Constant reminder of breast cancer	*“Numbness and disfigurement to breast, underarm, and nipple loss are a permanent reminder and affect intimacy, body image, and clothing choices.”*
Impact of long-term complications	*“I had no symptoms before diagnosis from a routine mammogram, and the surgery I had has left me in permanent discomfort and having to try and live with wearing a prosthetic, which I find unbearably uncomfortable.”*
*Reasons for avoiding specific procedures: Axillary node clearance*	
Avoiding unnecessary surgery	*“It would be wonderful if the lymph nodes did not have to be removed in order to establish whether they have cancer.”*
	*“By the time I had surgery, I had no detectable cancer left in my breast or lymph nodes, so I suspect I could have skipped at least ANC (axillary node clearance).”*
Long-term complications	*“I have after effects of it: lymphoedema and numbness still after 3 years.”*
	*“Removing the nodes has left me with a permanently vulnerable arm and lymphoedema. Managing my mastectomy (no reconstruction) is simple compared to dealing with the effects of node removal.”*
*Reasons for omitting radiotherapy*	
Short and long-term effects of treatment	*“I had acute radiotherapy cellulitis within hours of having my first radiotherapy treatment, so it made it even more unpleasant.”*
	*“Radiotherapy has caused permanent damage to my armpit and has hardened the implant causing ongoing pain.”“After 7 years, I was diagnosed with radiation-induced angiosarcoma. This resulted in further surgery.”*
Logistical issues—Stress of travelling for treatment/being away from family	*“Although the treatment was fine, I hated being away from my children Monday [to] Friday as I had to travel 4 hours for radiotherapy; therefore, I had to stay away from home.”*
	*“My reason for choosing radiotherapy is purely down to logistics as it was the only part of my treatment I had to travel for, 30-minute train journey, and 15-minute bus ride twice a day for fifteen days was absolutely exhausting.”*
Physical and psychological impact of treatment	*“I found the treatment very difficult and uncomfortable. I felt as if I was treated like a piece of meat, and certainly not a human being.… There needs to be a better system of delivering radiotherapy that thinks about the patient. I am still angry about my treatment eighteen months later.”*
	*“It was the hardest, physically and emotionally.”*

#### Chemotherapy

*“Chemo was by far the worst part of my treatment, worse than a simple mastectomy. Awful side effects: hair loss, fatigue, skin changes, neuropathy, mouth ulcers, and thrush to name a few. It’s systemic and time consuming and the side effects require a long recovery period.”*

Most women (*n* = 69, 35.6%) stated that they would have chosen to omit chemotherapy, with treatment described as brutal, barbaric, harsh, and rough with horrific and terrible side effects. The physical and psychological effects of treatment, including fatigue, nausea, and vomiting, coupled with fear of becoming seriously unwell and loss of identity were recurrent themes. Hair loss was a key issue; not only because it was distressing in itself, but also because it visibly identified women as being “a cancer patient.” Many respondents described how chemotherapy interfered with their ability to perform daily activities, including work, and disrupted their family life. Irreversible side effects, in particular peripheral neuropathy, which interfered with activities of daily living and hobbies, were reported as causing long-term problems (Table 2).

#### Endocrine Therapy

*“Taking this for 5–10 years is hard on the joints and bones, you have no sex life, and [it] is a constant reminder that you have had cancer.”*

Sixty-one women (31.4%) stated that they would choose not to take endocrine therapy if this was safe. Many cited side effects, specifically fatigue, joint pains, hair thinning, hot flushes, and weight gain as their rationale for this decision; several eloquently described how treatment impacted their well-being and quality of life (Table 2). Endocrine therapy was described as an “ageing” treatment that was a daily reminder for women of their breast cancer. The impact on women’s libido and relationships with their partner were key concerns. Concerns also were raised about the lack of support for women managing the side effects of their endocrine therapy and the expectation that they should just carry on (Table 2).

#### Surgery

*“I feel like this [surgery] has the greatest long-term effects, particularly psychologically.”*

Forty (20.6%) women stated they would have preferred to omit surgery. General comments related to the fact that surgery was “disfiguring” and had long-term impacts on their relationships and psychological well-being. Others would have liked to have avoided multiple operations to achieve clear margins, postoperative complications, or the need for a period of recovery following surgery.

Respondents repeatedly identified two procedures that they would specifically have chosen to avoid: mastectomy and axillary node clearance. The psychological impact of losing a breast was a key theme, together with the impact of surgery on body image, ability to dress freely, and relationships with partners. Some women reported long-term complications, including chronic pain that interfered with their ability to wear a prosthesis. Several women described long-term complications of axillary node clearance, including lymphoedema, and some expressed concerns about whether this extensive surgery was necessary for axillary staging (Table 2).

#### Radiotherapy

*“My reason for choosing radiotherapy is purely down to logistics as it was the only part of my treatment I had to travel for, 30-minute train journey and 15-minute bus ride twice a day for fifteen days was absolutely exhausting, plus radiotherapy caused problems with my implant, which resulted in 3 further operations and finally ending up flat on one side.”*

Fewest respondents (*n* = 20, 10.3%) opted to omit radiotherapy. The main reasons included avoidance of long-term complications, such as chronic pain and capsular contracture following implant reconstruction, and the unpleasant physical and psychological experiences of treatment. Several women described how having to travel daily for 3 to 5 weeks was challenging (Table 2).

#### Respondents’ Views of Reducing or Omitting Breast Cancer Treatments

*“We should be giving what can work—not just standard. We need to move to more personalised therapies.”*

A total of 151 women (64.3%) made additional comments related to safely reducing breast cancer treatments. The key themes are summarized in Table 3.

**Table 3:** Other considerations related to breast cancer treatment de-escalation

Theme	*Example respondent quotes*
Support for a more personalised approach to breast cancer treatments	*“It is very good news that there is potential for the treatment to be more tailored to the individual. I was diagnosed 11 years ago and was just told you are having x, y, & z treatment. There was no discussion or explanation as to how that decision was reached.”*
	*“I think it is important to be able to offer women less aggressive or reduced treatment in the future, but factors, such as individual risk tolerance, tolerance for medication, anxiety, fear of death, family, children, individual life station, should always be taken into consideration and may mean that reduced treatment is not right for everyone. Less is not always more. Reduced treatment is a good option if it best meets a woman’s needs, but this has to be decided patient by patient.”*
Prioritisation of survival and reducing risk of recurrence	*“I felt that I wanted everything they could give; I wanted every chance not to have it return. I’m afraid of it returning.”*
	*“I think that rather than treatment being reduced, it should be treated as aggressively as possible, via the treatments from which people are most likely to benefit.”*
	*“Being diagnosed <50 years with stage 3 (locally advanced/grade 3) cancer and with two young teenagers survival is my absolute priority. But it would be good to have less side effects, particularly long-term side effects, which have significantly affected my return to work and in turn significantly affected our financial situation.”*
Possible guilt/regret if opted for less treatment and developed recurrence	*“I would be totally against reducing treatment as patients like to feel they have had all available treatment to prevent a) death and b) spread. If either event occurs and they have not had the benefit of all available treatment patients are devastated and understandably will think that the event could have been prevented.”*
	*“Personally for me, I will take any and as much treatment as is recommended, I never want to be in a position where I wonder would I have gotten more time if I had taken the additional treatment.”*
Need to research to show that reducing treatments is safe	*“I would only want my treatment reduced if research showed it would not be detrimental to my prognosis.”*
	*“The research would have to be rock solid, as I would need to know from a psychological perspective that the treatment definitely would not help me at all.”*
	*“I would be reticent to remove treatment unless the really long-term survival data were there to support. We seem to focus a lot on 5-year survival rates, but as a woman in my early 50s, I am interested in the 20+-year survival data just as much as 5-year. I wouldn’t want to remove something that increased the recurrence risk later down the line.”*
Need for better communication about the absolute benefits of individual treatments to support women to make more informed decisions about whether they are needed	*“patients currently think that MORE is better…that the more they do, the better outcome they will have…it needs to be clearly indicated to them, in terms they can understand, that perhaps LESS can be JUST as effective…but this information must be presented in language that they understand with perhaps analogies that they can clearly understand in order for them to make an educated decision…they may still make the same decision…but the decision will be theirs”*
	*“Saying radiation will reduce recurrence by 50%, but don’t tell you that in your case the probability of recurrence is only 1%, so is it worth the long-term negative affects for 0.5% improvement? Of course not! But few women actually ask what the actual numbers are for them. IMO the doctor should be very open about the chance of recurrence for them, not pushing a procedure because it reduces the chance by 50 or 20 or whatever %.”*

Overall, respondents were supportive of personalized approaches to breast cancer treatments with a focus on reducing side effects, toxicity, and avoidable morbidity and improving quality of life. Many women, however, highlighted that survival and reduction of breast cancer recurrence was their absolute priority. Several expressed concern that they may have experienced feelings of regret or guilt if they had reduced treatment and subsequently developed metastatic disease. Women clearly stated that there was a need for high-quality research to show that reducing treatment was safe before this approach would be acceptable to patients. The need for accurate communication of the absolute benefits of specific treatments also was a key theme with some women, suggesting that this information should be routinely shared to help patients make informed decisions regarding whether the benefits of any specific treatment justified the associated risks (Table 3).

## Discussion

Optimizing breast cancer treatment with risk-adapted approaches is research priority, with multiple, ongoing studies exploring the possibility of safely reducing or omitting components of treatments to reduce treatment burden, minimize morbidity, and improve quality of life.^3^ This international survey of the experiences of women treated for early breast cancer and their attitudes to the safe omission of components of their treatment highlights the importance of these studies and the need for ongoing work to improve personalization of care. Women’s responses clearly highlight that all components of breast cancer treatment have significant and lasting effects on individuals’ well-being and quality of life, impacting their relationships, family lives, ability to work, and sense of self. Different respondents reported choosing to omit different parts of their treatment, highlighting that these decisions are highly individual and based on personal circumstances and preferences. Furthermore, whilst there was broad support for reducing treatments that had little or no benefit, breast cancer survival was an absolute priority and respondents clearly highlighted the need for high-quality research to demonstrate the safety of this approach as well as the need for effective communication about the absolute risks and benefits of proposed treatments to allow them to make fully informed decisions regarding their options.

This is, to our knowledge, the largest international survey to explore the attitudes of individuals with lived breast cancer experience to the safe omission of components of their treatment. Perhaps not unexpectedly, chemotherapy was the treatment most women would choose to omit if this was safe, not only due to short-term side effects but also longer-term toxicities, such as peripheral neuropathy and the psychological impact of treatment. These findings highlight the importance of ongoing studies evaluating whether gene expression profiling tests can be used to select patients with node-positive estrogen receptor-positive HER-2–negative breast cancer who are unlikely to benefit substantially from chemotherapy.^4^ However, there also is a clear need to develop new studies that explore the possibility of safely reducing chemotherapy in other disease subtypes.^14^

More than a quarter of women would choose to omit endocrine therapy, mainly because of side effects and the impact of estrogen deprivation on quality of life and well-being. Despite these findings, currently very few studies are focused on de-escalation of endocrine therapy. Only two biomarker-driven prospective studies evaluated the outcomes of shorter duration of treatment in low-risk patients.^8,15^ Recent work, however, suggests that it may be possible to use gene-expression profiling to identify patients who may not benefit from endocrine therapy, raising the possibility of future personalized treatment approaches to this issue.^16–18^ This survey confirms that there is an urgent need for research to optimize use of adjuvant endocrine therapy, potentially allowing some patients to avoid the known toxicities of this treatment as well as to develop strategies to appropriately support women in whom treatment would be beneficial.^19^

Fewer women opted to omit surgery, but for some, this clearly had a profound and long-lasting impact on their well-being and quality of life with mastectomy and axillary node clearance highlighted as two procedures that women would choose to avoid. Mastectomy rates remain high in the United Kingdom; up to 40% of all women with breast cancer undergo this procedure.^20^ However, many of these may be avoidable with appropriate use of neoadjuvant systemic treatments and oncoplastic techniques. Research into strategies to optimize use of, and access to, these approaches is thus urgently needed to improve outcomes for patients. Avoidance of axillary node clearance in women with nodal involvement has already been identified as the top breast cancer surgery research priority in the United Kingdom and the breast cancer community worldwide.^3,21^ Studies are planned and ongoing to explore whether ANC can be safely omitted in selected patient groups.^22,23^ Trials of minimally invasive surgical approaches for the treatment of smaller, good prognosis tumors and the complete omission of surgery in patients who have a complete response to neoadjuvant chemotherapy are ongoing, offering future patients the potential of less radical options for surgery to the breast.^24–27^

Despite the large number of ongoing international studies focused on the omission of radiotherapy, only 10% of women would choose to omit this treatment.^5–7^ Furthermore, the main rationale for this choice, specifically the logistical issues in attending for treatment may now be largely historic given widespread adoption of hypofractionated radiotherapy regimens requiring only 5 days of treatment for many patients.^28^ Receipt of radiotherapy is no longer perceived as a burdensome treatment, and findings of this international survey suggest that researchers should now focus greater effort on developing studies to reduce the intensity and treatment burden of other treatment modalities.

This survey provides valuable information on patients’ priorities for reducing breast cancer treatments, but the design has limitations. Participants completing the survey, for example, are a self-selected group who may have particularly good (or bad) experiences of treatment, such that these findings may not be generalizable to breast cancer patients more broadly. The resonance of these findings with previous surveys and clinical practice, together with the inclusion of a geographically diverse group of individuals who had received a range breast cancer treatments, however, suggests that this is unlikely.^11^ There is an underrepresentation of minority ethnic groups, and the views expressed may not reflect the concerns of these women. Furthermore, the median year of treatment reported by respondents was 2018. It is possible that some of the issues raised are no longer relevant in an era of more contemporary treatment. With the exception of the introduction of hypofractionated radiotherapy, however, there have been few significant treatment advances in early breast cancer in the intervening years.^28^ The concerns and issues expressed by women in this survey will still be largely applicable and form an excellent basis for the development of future studies to address areas of importance to patients.

While there was widespread support for a more personalized, risk-adapted approach to breast cancer treatments, many survey respondents highlighted that this needed to be underpinned by high-quality robust research to support the long-term safety of this approach. Development of a broader portfolio of studies, co-designed with patient partners is needed to allow safe de-escalation of a range of treatments in a way that is acceptable and meaningful to patients. The key to this will be the development of strategies to promote effective communication of risk and optimize informed consent, not only to facilitate future clinical trials but also as part of routine shared decision making to allow women to judge for themselves whether the absolute benefits of treatment justify the risks. Adaptation of interventions to optimize informed consent in standard randomized trials may be one way this could be achieved.^29^ Furthermore, there is a need for a standardized terminology for research in this area. As highlighted in this study, the concept of “reducing” treatments is inherently concerning to patients and similarly terms, such as “de-escalation,” although used routinely in clinical research,^14^ carry a potential implication of withdrawal of treatments. Framing future research as risk-adapted or personalized treatment studies may mitigate against this connotation and communicate the concept that treatment intensity should be commensurate with an individual’s level of risk. Qualitative interviews, however, are needed to allow patients’ views of such terminology to be explored so that future studies can be framed in a way that is most acceptable and comprehensible to patients.

The majority of patients will now be long-term breast cancer survivors, so the short- and long-term effects of treatment on women’s well-being and quality of life matter.^30^ Reducing avoidable side effects and toxicity must be a research priority. This survey highlights that future studies should be patient-focused and developed and designed to answer questions that are relevant and clinically meaningful to breast cancer patients, support informed treatment decision-making, and ensure the best possible outcomes for each individual.
